# Transplacentally Acquired Maternal Antibody against Hepatitis B Surface Antigen in Infants and its Influence on the Response to Hepatitis B Vaccine

**DOI:** 10.1371/journal.pone.0025130

**Published:** 2011-09-22

**Authors:** Zhiqun Wang, Shu Zhang, Chao Luo, Qianzhen Wu, Qilan Liu, Yi-Hua Zhou, Yali Hu

**Affiliations:** 1 Department of Obstetrics and Gynecology, Nanjing Drum Tower Hospital, Nanjing University Medical School, Jiangsu, China; 2 Jiangsu Family Planning Institute, Nanjing, Jiangsu, China; 3 Departments of Laboratory Medicine and Infectious Diseases, Nanjing Drum Tower Hospital, Nanjing University Medical School, Jiangsu, China; 4 Jiangsu Key Laboratory for Molecular Medicine, Nanjing University Medical School, Jiangsu, China; Duke University School of Medicine, United States of America

## Abstract

**Background:**

Passively acquired maternal antibodies in infants may inhibit active immune responses to vaccines. Whether maternal antibody against hepatitis B surface antigen (anti-HBs) in infants may influence the long-term immunogenicity of hepatitis B vaccine remains unknown.

**Methodology/Principal Findings:**

Totally 338 pairs of mothers and children were enrolled. All infants were routinely vaccinated against hepatitis B based on 0-, 1- and 6-month schedule. We characterized the transplacental transfer of maternal anti-HBs, and compared anti-HBs response in children of mothers with or without anti-HBs. In a prospective observation, all 63 anti-HBs positive mothers transferred anti-HBs to their infants; 84.1% of the infants had higher anti-HBs concentrations than their mothers. One and half years after vaccination with three doses of hepatitis B vaccine, the positive rate and geometric mean concentration (GMC) of anti-HBs in 32 infants with maternal anti-HBs were comparable with those in 32 infants without maternal antibody (90.6% *vs* 87.5%, *P* = 0.688, and 74.5 *vs* 73.5 mIU/ml, *P* = 0.742, respectively). In a retrospective analysis, five and half years after vaccination with three doses vaccine, the positive rates of anti-HBs in 88 children of mothers with anti-HBs ≥1000 mIU/ml, 94 children of mothers with anti-HBs 10–999 mIU/ml, and 61 children of mothers with anti-HBs <10 mIU/ml were 72.7%, 69.2%, and 63.9% (*P* = 0.521), respectively; anti-HBs GMC in these three groups were 38.9, 43.9, and 31.7 mIU/ml (*P* = 0.726), respectively.

**Conclusions/Significance:**

The data demonstrate that maternal anti-HBs in infants, even at high concentrations, does not inhibit the long-term immunogenicity of hepatitis B vaccine. Thus, current hepatitis B vaccination schedule for infants will be still effective in the future when most infants are positive for maternal anti-HBs due to the massive vaccination against hepatitis B.

## Introduction

Hepatitis B vaccine is highly effective in preventing hepatitis B virus (HBV) infection. Antibodies directed against hepatitis B surface antigen (anti-HBs) after vaccination with three doses vaccine may maintain more than two decades in most vaccinees [1], [2]. As at December 2008, 177 countries included the hepatitis B vaccine into their national infant immunization programs [3]; all infants in these countries may be vaccinated with the vaccine. Currently, around 30–40% of the child-bearing age women in China are positive for anti-HBs as a result of vaccination or natural infection [4]. Predictably, most child-bearing age women worldwide will be positives in the future due to the mass vaccinations. Accordingly, their infants will also be positive for anti-HBs because of the transplacental transfer of maternal IgG.

Transplacentally acquired maternal antibodies protect infants against infections in the early period of life. On the other hand, maternal antibodies in infants may also interfere with the active immune responses to vaccines against various pathogens, including measles, poliovirus, hepatitis A virus, pertussis, tetanus and diphtheria toxoids, and others [5]–[11]. Previously, we found that, after the second dose vaccine, the antibody response to hepatitis B vaccine in infants with maternal anti-HBs was lower than that in infants without the maternal antibody, and the short-term immunogenicity of hepatitis B vaccine in infants with high maternal anti-HBs (>1000 mIU/ml) was significantly inhibited after vaccination with the third dose [12]. In this report, we characterized the transplacental transfer of anti-HBs in infants and measured the anti-HBs levels in children, who had been vaccinated against hepatitis B in the presence or absence of maternal anti-HBs during infantile period, to clarify whether the long-term immunogenicity of hepatitis B vaccine is impaired by the maternal anti-HBs.

## Materials and Methods

### Study subjects

**Figure 1:**
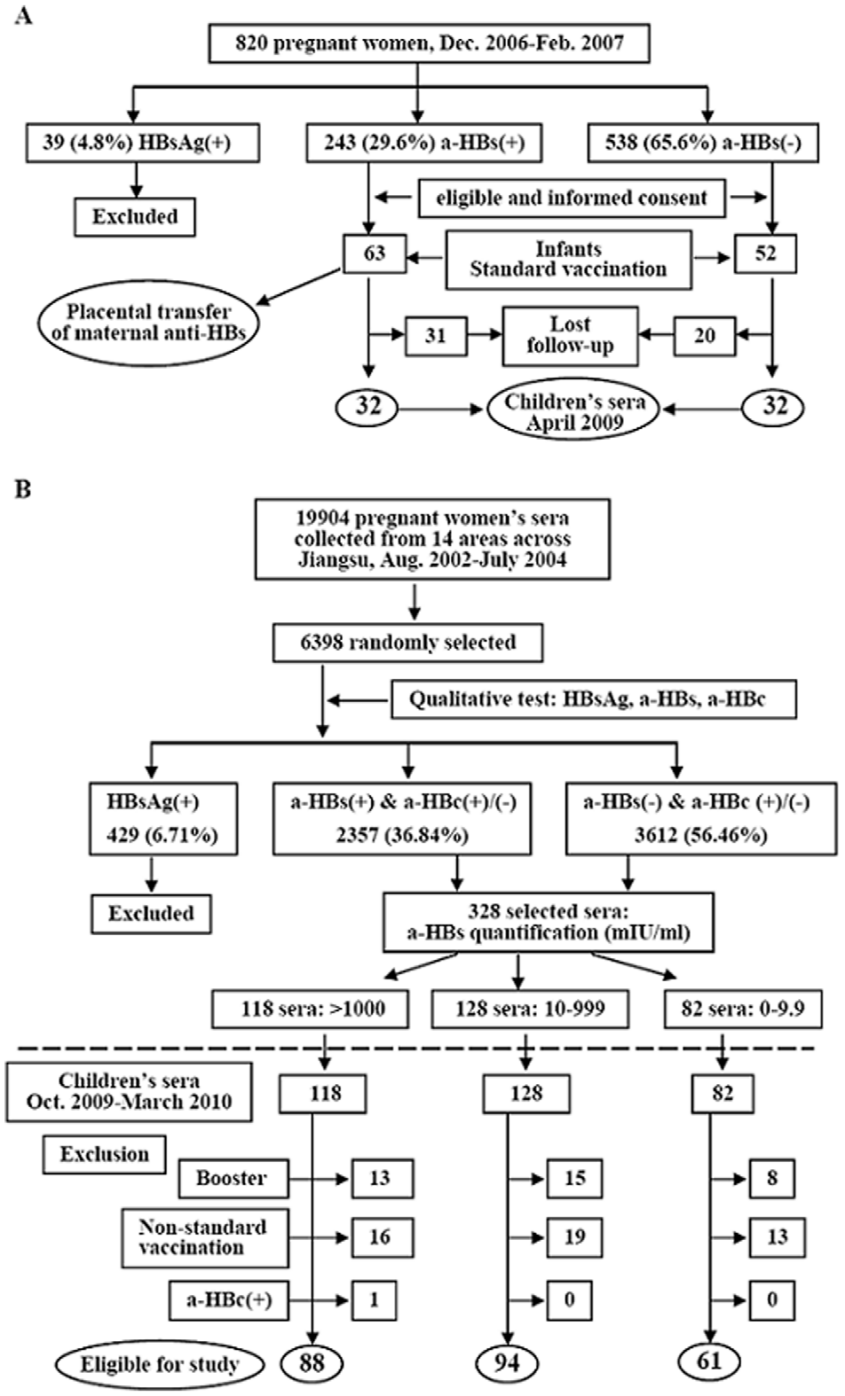
Flow diagram of study subjects. A, prospective cohort; B, retrospective cohort. HBsAg, hepatitis B surface antigen; a-HBs, antibody against HBsAg; a-HBc, antibody against hepatitis B core antigen.

The subjects recruited in this investigation included two groups (Figure 1). One group was prospectively studied (Figure 1A). The pregnant women (totally 820) attending the antenatal care unit at Nanjing Drum Tower Hospital between December 2006 and February 2007, were routinely screened for hepatitis B surface antigen (HBsAg), anti-HBs, and antibody against hepatitis core antigen (anti-HBc). Those who were positive for anti-HBs but negative for HBsAg, or were negative for all the three markers were explained the project and invited to participate. The women with chronic diseases such as diabetes mellitus, asthma, HIV infection, syphilis, and autoimmune diseases were excluded. Finally, 63 anti-HBs positive mothers and their singleton newborn infants were enrolled in analysis of anti-HBs placental transfer, and the 63 infants were assigned into the group with maternal anti-HBs. Concurrently, 52 women with negative for all the three markers and without above chronic diseases consented to participate and their infants were assigned into the group of children without maternal anti-HBs.

Blood samples were collected from the women immediately before or during delivery and from their newborns immediately after birth by umbilical artery sampling. Of above infants, 32 were followed with anti-HBs and other HBV serologic markers in April 2009. As controls, 32 infants of mothers without anti-HBs were similarly followed. All infants were vaccinated with the same vaccine lot (5 µg yeast recombinant HBsAg per dose) according to the recommended schedule, i.e, three doses, within 24 hours, 1 month, and 6 months respectively after birth as previously described [12]. The vaccine, which has been licensed by Merck & Dohme Co, was manufactured by Shenzhen Kangtai Biological Company, China.

Another group was retrospectively investigated (Figure 1B). In a study on the provincial prevalence of birth defects conducted August 2002 to July 2004 [13], serum samples from 19 904 pregnant women aged 20–42 years, at gestation of 15–20 weeks, from 8 counties (rural area) and 6 cities (urban) in Jiangsu Province, China, were collected and stored at −30°C. These women had been selected to be the representatives of the pregnant women population in Jiangsu and all delivered their infants in hospitals. Recently, we retrospectively measured the HBV serologic markers in the 6398 sera, which were randomly selected from above samples, and found that 2357 (36.84%) of them were positive for anti-HBs [4]. According to the anti-HBs status of women during their pregnancy, we followed 328 pairs of mothers and children and collected children's blood samples during October 2009 through March 2010. HBsAg was negative in all 328 children, in whom 48 were not vaccinated as the recommended schedule, 36 received booster immunization, and one experienced a natural infection because of the presence of anti-HBc. Thus, we excluded these 85 children and finally included 243 children in the observation, of whom 88, 94, and 61 were born to mothers with anti-HBs ≥1000, 10–999, and <10 mIU/ml, respectively (Figure 1B). Among the 61 mothers with anti-HBs <10 mIU/ml, 24 were totally absence of anti-HBs, and 13, 10, and 14 had the antibody <2, 2.1–4.9, and 5–9.9 mIU/ml, respectively. Because the mass vaccination in China is administered by each Provincial Health Department, all the hepatitis B vaccine used in this group children was from the same manufacturer as used in the prospective group and had equal amount of recombinant HBsAg (5 µg) in each dose.

This study was approved by the institutional review boards of Nanjing Drum Tower Hospital and Jiangsu Family Planning Institute. Written Informed consent was obtained from each woman in the prospective cohort; the infant/child's consent was assigned by his/her mother. In the retrospective cohort, the mothers consented to participate in the birth defect study conducted from August 2002 to July 2004 [13] and their serum samples were used in this study; the mothers consented to be interviewed and consented to their children's participation in the following-up during October 2009 through March 2010. All infants were also administered with other routine vaccinations as recommended by China Expanded Program on Immunization.

### Assays for HBV serologic markers

Qualitative tests of HBsAg, anti-HBs, and anti-HBc in serum were performed with commercial enzyme immunoassay (EIA) kits (Huakang Biotech, Shenzhen, China). The commercial microparticle enzyme immunoassays (AxSYM AUSAB, Abbott, North Chicago) were used for quantitatively determining anti-HBs and anti-HBc. All samples were tested by the two technicians who were unaware of the serum identity. Paired maternal and neonatal sera were analyzed in parallel. When anti-HBs level was beyond the upper detection limit (1000 mIU/ml), the sera were retested by dilution with 20% bovine fetal serum in phosphate-buffer saline (PBS).

### Analysis of anti-HBs IgG subclasses

Analysis of the IgG subclasses of anti-HBs was performed by an EIA as previously reported [14], [15] with minor modifications. Anti-HBs positive sera were 5-fold diluted and added to microtiter plate wells coated with recombinant HBsAg (Huakang Biotech). After incubation and washes, mouse anti-human IgG subclass specific monoclonal antibodies (0.1 µg/well in 100 µl), including IgG1, IgG2, IgG3 (Invitrogen, Carlsbad, CA), and IgG4 (clone HO6023, AbD Serotec, Oxford, UK) were used to probe the subclasses of anti-HBs respectively. The activity of anti-HBs specific for each of the four IgG subclasses was expressed as the OD450 of samples.

### HBsAg inhibition test

Binding of anti-HBs to HBsAg, a surrogate test for in vitro neutralization, was performed as reported elsewhere [16], [17] with modifications. An HBsAg-positive serum from a hepatitis B patient was titrated and 50-fold diluted in PBS based on the preliminary experiment results. The 25 µl aliquot of the diluted serum was mixed with equal volume of each of the paired maternal and neonatal sera (including negative controls) at room temperature for 1 h. The mixture was then subjected to detection of the HBsAg with the EIA kit (Huakang Biotech), and the OD450 was read on the ELISA reader. The inhibition was expressed by a percentage calculated by the following formula: ([OD450 of negative control−OD450 of anti-HBs positive sample]/OD450 of negative control)×100.

### Statistical analysis

Data were analyzed using SPSS version 13 (SPSS Inc., Chicago, USA). Correlations between maternal and cord blood titers were derived with linear regression analysis. Statistical comparisons of continuous variables were analyzed by *t*-test (comparison between two groups) or one-way ANOVA test (comparison among three groups) and a *χ* ^2^ test was used to analyze and compare categorical data. Anti-HBs ≥10 mIU/ml was defined to be positive. When compared among different subgroups, the anti-HBs level was expressed by geometric mean concentration (GMC) followed by minimum and maximum values. When anti-HBs in a serum was absolutely absent, the sample was considered to have 0.01 mIU/ml in calculating anti-HBs GMC. The Mann-Whitney *U* and Kruskal-Wallis rank-sum tests were used for comparisons of GMC between subgroups. A *P* value of <0.05 was considered statistically significant.

## Results

### Transplacental transfer of maternal anti-HBs

**Figure 2:**
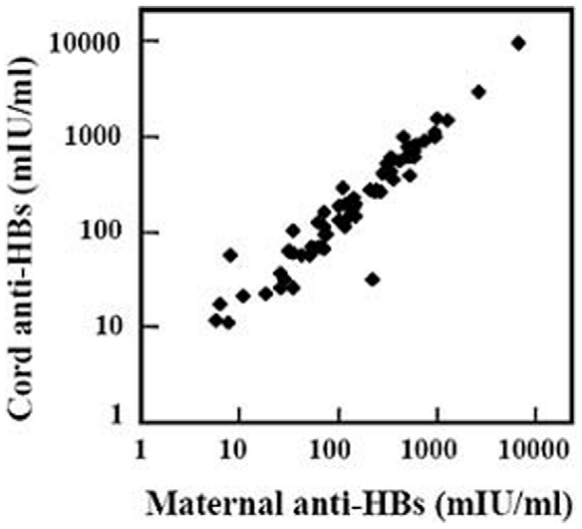
Correlation of transplacentally transferred anti-HBs in infants with the maternal antibody (linear regression analysis, *y* = 1.393*x*−37.286, r = 0.992, *P*<0.001, n = 63).

Transplacental transfer of maternal anti-HBs was analyzed in 63 pairs of anti-HBs positive mothers and their infants. The mothers were 20–36 years of age (mean, 27.2±2.8). Of these mothers, 41 had been vaccinated against hepatitis B, 21 had not been vaccinated, and the remaining one was uncertain. All of the 21 unvaccinated women were positive for anti-HBc, indicating that they acquired the specific immunity by resolved HBV infection. All full-term infants were healthy (Apgar scores >8) at birth with body weights 2800–4600 g (mean, 3354.7±369.6). The male and female infants were 28 and 35 respectively. Each of the 63 mothers transferred the anti-HBs to her infant because anti-HBs was positive in each cord serum. To analyze the transplacental transfer efficiency, we compared the anti-HBs concentrations between maternal and cord sera. As shown in Table 1, 84.1% of the cord serum samples had higher concentrations than the corresponding maternal sera. Figure 2 shows the significant positive correlation between maternal and cord blood anti-HBs (linear regression analysis, *y* = 1.393*x*−37.286, r = 0.992, *P*<0.001). Infants' sex, the delivery modes, and mothers' ages were not correlated with the efficiency of transplacental anti-HBs transfer (data not shown).

**Table 1:** Comparison of anti-HBs between infants and mothers.

Ratio of cord/maternal anti-HBs	No. of paired sera (%)
>1.50	21 (33.3)
1.01–1.49	32 (50.8)
1.00	2 (3.2)
0.50–0.99	8 (12.7)
Total	63 (100.0)

### IgG subclass profiles of anti-HBs

**Figure 3:**
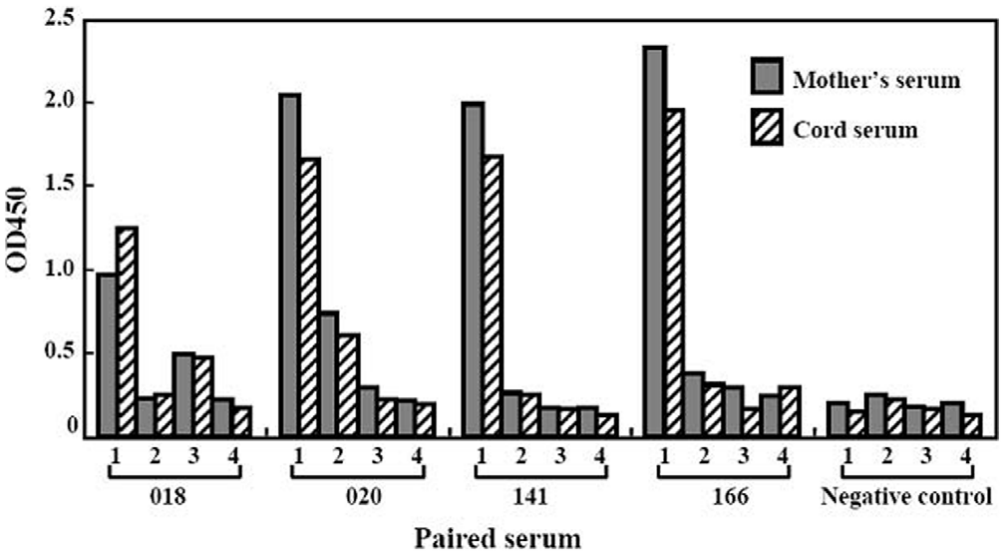
Anti-HBs IgG subclasses in paired mother-infant sera. 1, 2, 3, and 4 under the X axial indicate the subclasses IgG1, G2, G3, and G4, respectively. Sera 018 and 020 were from mothers recovered from natural HBV infection, while sera 141 and 166 were from vaccinated mothers. The data from 4 paired sera were used to represent anti-HBs IgG subclasses distribution in 20 paired mother/infant sera.

Since anti-HBs in most of the infants was higher than that in their mothers (Table 1), and IgG1 is the most efficiently transplacentally transported subclass [18], we attempted to clarify which subclass of anti-HBs IgG was predominant in the infants. We selected 20 paired mother-infant sera (11 vaccinated, 8 not vaccinated, and 1 uncertainty of the vaccination) and analyzed the anti-HBs IgG subclasses. Anti-HBs IgG1 subclass was predominant in all these samples, regardless of the antibody induced by vaccination or by resolved HBV infection, and three other IgG subclasses accounted for minor portion of the total anti-HBs. Figure 3 shows the representative results of the four paired sera.

### Binding of HBsAg by maternal anti-HBs

**Figure 4:**
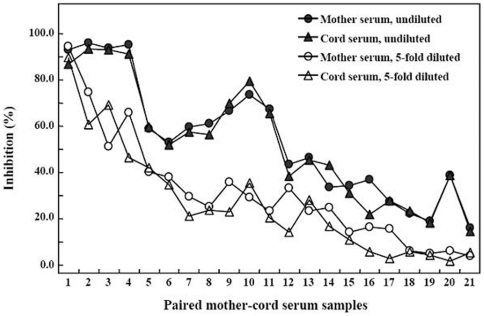
Neutralization of HBsAg by paired mother-infant sera. An HBsAg positive serum from a hepatitis B patient was preincubated with each of paired sera, and the unneutralized HBsAg was detected with enzyme immunoassay. The inhibition (%) was calculated by the following formula: ([OD450 of HBsAg preincubated with anti-HBs negative serum−OD450 of HBsAg preincubated with anti-HBs]/OD450 of HBsAg preincubated with anti-HBs negative serum)×100. Anti-HBs titers in mother serum samples 1–21 were 6613, 2644, 992, 944, 611, 565, 492, 369, 352, 318, 273, 244, 149, 148, 116, 73, 44, 36, 29, 18, and 16 mIU/ml, respectively; those in cord serum samples 1–21 were 9663, 2870, 1568, 1106, 798, 766, 616, 348, 590, 525, 255, 271, 194, 147, 291, 67, 57, 103, 31, 22, and 20 mIU/ml, respectively.

Since an efficient in vitro cell culture system is not available for testing the neutralizing activity of anti-HBs, we used a surrogate assay to test the inhibition of HBsAg by maternal anti-HBs in the cord blood. Concurrently, the neutralization of HBsAg by the mothers' sera was also tested. We measured the inhibition of 21 paired mother-infant sera including those containing the highest and lowest concentrations of anti-HBs in the samples. Figure 4 shows that all 21 undiluted paired sera inhibited the reactivity of HBsAg, more than 95% by the sera containing high level anti-HBs (paired samples 1–4) and nearly 20% by the sera having the low titers. When the sera was 5-fold diluted, the inhibition also decreased, except for the paired samples 1, in which 5-fold diluted sera still had the antibody >1000 mIU/ml because of their high levels of anti-HBs (6613 and 9663 mIU/ml in mother's and cord's sera respectively); the data demonstrate the specificity of the assay. Generally, the inhibition tended to be in a dose-dependent manner although some samples containing relatively low concentrations had greater inhibition rates. Noticeably, the cords' blood usually had higher levels than mothers' blood (Table 1 and Figure 2); however, the inhibition rates of the paired sera were comparable.

### Anti-HBs levels in children vaccinated in the presence or absence of maternal anti-HBs

To clarify whether maternal anti-HBs in infants may impair the long-term immunogenicity of hepatitis B vaccine, we prospectively measured anti-HBs in 32 children of mothers with positive anti-HBs and in 32 children of mothers with negative anti-HBs around one and half years after the infants had been vaccinated with three doses vaccine. The general characteristics of mothers and their infants were comparable in the two subgroups (Table 2). The positive rates of anti-HBs were 90.6% and 87.5% respectively (*χ* ^2^ = 0.161, *P* = 0.688), and the anti-HBs GMC levels were also comparable (74.5 *vs* 73.5 mIU/ml, Mann-Whitney U test, *Z* = −0.282, *P* = 0.742) (Table 2).

**Table 2:** Anti-HBs titers in children after vaccination with three hepatitis B vaccine doses.

	Anti-HBs in cord serum (mIU/ml)		Statistical analysis	
	≥10 (n = 32)	0 (n = 32)	Method	*P*
Mothers' age at delivery (year)	26.87±2.54	27.03±2.56	*t*-test, *t* = 0.249	0.804
Gestational age (week)	39.21±1.08	39.47±1.33	*t*-test, *t* = 0.750	0.457
Birth weight (g)	3423.16±459.37	3360.48±343.86	*t*-test, *t* = 0.550	0.585
Male/Female	17/15	14/18	*χ* ^2^ test, *χ* ^2^ = 0.563	0.453
Delivery modes			*χ* ^2^ test, *χ* ^2^ = 0.075	0.784
Cesarean section	9	10		
Spontaneous labor	23	22		
Children's age	2.16±0.08	2.17±0.06	*t*-test, *t* = 0.914	0.364
Anti-HBs+ No. (%)	29 (90.6)	28 (87.5)	*χ* ^2^ test, *χ* ^2^ = 0.161	0.688
Anti-HBs (mIU/ml)^a^	74.5 (6.1–884.0)	73.5 (3.1–930.2)	Mann-Whitney U test, *Z* = −0.329	0.742

a Anti-HBs level was expressed in geometric mean concentration followed by minimum and maximum values in parenthesis.

To investigate whether high titer of maternal anti-HBs in infants may have long-term influence on the immune response to hepatitis B vaccine, we further compared anti-HBs response in children with different levels of maternal anti-HBs. Of the 32 infants of anti-HBs positive mothers, 26 had maternal anti-HBs 31.5–895.0 mIU/ml and 6 had the antibody 1001.0–9663.0 mIU/ml in their cord blood. At approximately one and half years after the three doses vaccine, the rates of positive anti-HBs in above subgroups were 92.3% (24 of 26) and 83.3% (5 of 6) respectively (*P* = 0.525), and the anti-HBs GMT was comparable (74.2 *vs* 81.7 mIU/ml, *P* = 0.562).

Since the number of infants with high titers (>1000.0 mIU/ml) of maternal anti-HBs was small (6 infants), follow-up period was relatively short (one and half years), and the drop out rate was high in the prospective cohort (Figure 1A), we considered that data from larger cohort of subjects with longer follow-up period would validate the results of the prospective study. Thus, we further compared the anti-HBs levels in more vaccinated children born to mothers who had high (≥1000 mIU/ml) and moderate to low (999–10 mIU/ml) anti-HBs or who were absence or almost absence of anti-HBs (0–9.9 mIU/ml) during the pregnancy in a retrospective survey. In the three subgroups, the mothers' ages at delivery were similar and positive rates of anti-HBc were comparable (Table 3). Table 3 also shows that at around five and half years after completion of the vaccination, the positive rates of anti-HBs in children in these three subgroups were 72.7%, 69.2%, and 63.9% (*χ* ^2^ = 1.505, *P* = 0.521) respectively, and the GMC titers of anti-HBs in these children were 38.9, 43.9, and 31.7 mIU/ml respectively (Kruskal-Wallis rank-sum test, *χ* ^2^ = 0.630, *P* = 0.726).

**Table 3:** Long-term immune response after vaccination against hepatitis B in children born to mothers with various anti-HBs levels.

	Anti-HBs in mothers during pregnancy (mIU/ml)			Statistical analysis	
	≥1000 (n = 88)	10–999 (n = 94)	<10 (n = 61)	Method	*P*
Maternal age	25.22±3.53	24.82±3.95	25.77±3.23	One-way ANOVA test, *F* = 0.752	0.282
Children's age	6.14±0.49	6.04±0.57	5.83±0.67	One-way ANOVA test, *F* = 5.071	0.007
Male/Femal	50/38	51/43	37/24	*χ* ^2^ test, *χ* ^2^ = 0.618	0.734
Anti-HBc+ No. (%)	10 (11.36)	11 (11.70)	2 (3.28)	*χ* ^2^ test, *χ* ^2^ = 3.644	0.162
Anti-HBs+ No. (%)	64 (72.73)	65 (69.15)	39 (63.93)	*χ* ^2^ test, *χ* ^2^ = 1.305	0.521
Anti-HBs (mIU/ml)^a^	38.90 (0–15000.0)	43.87 (0–5886.9)	31.65 (0–12452.9)	Kruskal-Wallis rank-sum test, *χ* ^2^ = 0.630	0.726

a Anti-HBs level was expressed in geometric mean concentration followed by minimum and maximum values in parenthesis.

## Discussion

Our study confirms that anti-HBs in pregnant women may efficiently transfer into their fetuses and the maternal anti-HBs in the majority of newborns is higher than that in their mothers. Furthermore, our findings demonstrate that the maternal anti-HBs, even at high concentrations, in infants does not inhibit the long-term immunogenicity of hepatitis B vaccine.

Placental transfer of IgG is an active process and requires the binding of maternal IgG and neonatal Fc receptors in the placenta [19]. As a result, the maternal antibodies in full-term newborns are usually higher than those in their mothers [18]. In accordance with this, we found that anti-HBs in most cord sera exceeded the maternal levels (Table 1 and Figure 2). This may be attributed to the predominance of anti-HBs IgG1 subclass (Figure 3), since IgG1 is the most efficiently transferred immunoglobulin subclass [18]. We also found that anti-HBs, induced either by natural infection or by vaccination, was IgG1 predominance, which is in agreement with the previous findings [15]. Therefore, as expected, anti-HBs positive pregnant women, regardless of how the antibody is induced, can efficiently transfer anti-HBs to their infants. In contrast to our findings, in 60 pregnant Nigerians, transplacental transfer of anti-HBs occurred only in 59% of the infants [20]. At the moment, it is difficult to explain the difference. The transfer of IgG antibody against measles virus from mothers to their infants in Nigeria is also less efficient compared with that in German women [21]. Thus, other coincident factors may also influence the antibody transplacental transfer in Nigerians.

In this study, we compared the long-term immunogenicity of hepatitis B vaccine in the retrospective group children based on their mothers' anti-HBs levels during pregnancy (Table 3), rather than based on the anti-HBs levels in the children immediate after birth. However, since the mothers' anti-HBs level was well correlated with that in the infants (Figure 2), the comparability of anti-HBs positive rates and of GMC levels among the different children subgroups (Table 3) was unlikely caused by the grouping. Furthermore, the prospective survey showed that, one and half years after three doses vaccine, the positive rate and GMC level of anti-HBs were each similar to those in children with or without maternal anti-HBs respectively (Table 2). Additionally, we found only one natural booster, which was excluded in the analysis, among children in the retrospective group and did not find any natural booster in the prospective group, indicating that anti-HBs in all children is the result of the vaccination. Therefore, the data in the present study are sufficient to conclude that the long-term immunogenicity of hepatitis B vaccine in infants is not inhibited by the maternal anti-HBs.

Previously, we found that the short-term immune response to hepatitis B vaccine in infants is impaired by high titer maternal anti-HBs [12]. In this study, we demonstrate that the long-term immune response to hepatitis B vaccine is not influenced by the maternal anti-HBs, even at the high concentrations. This appears to be somewhat unusual since the immunogenicity of many other vaccines is to some extent blocked by the maternal antibodies [5]–[11]. The mechanisms by which maternal antibodies influence infant vaccine responses involves: (1) neutralization of live viral vaccines, (2) inhibition of infant B cell activation by Fcγ-receptor mediated signals, (3) elimination of maternal antibody-antigen complex through Fc-dependent phagocytosis, and (4) epitope masking by maternal antibody, preventing antigen binding by the B cells [6]. The hepatitis B vaccine is composed of the recombinant HBsAg. Quantitatively, the maternal anti-HBs in infants is in much excess of the vaccine HBsAg since one dose vaccine usually contains only 5 or 10 µg HBsAg. Theoretically, because the antibody in infants may bind to the HBsAg (Figure 4), the vaccine HBsAg should be neutralized by maternal anti-HBs, resulting in reduced immunogenicity of hepatitis B vaccine through one or more above mechanisms. Indeed, the immune response to two doses vaccine in infants with maternal anti-HBs is lower than that in infants without maternal anti-HBs [12]; however, this inhibition is completely overcome by the third immunization in infants with moderate to low maternal anti-HBs [12], indicating that the inhibition is transient. Actually, early reports show that high-dose hepatitis B immune globulin (anti-HBs) may transiently impair the immune response to hepatitis B vaccine [22], [23]. Thus, the data support that priming of humoral immune response to the vaccine HBsAg is not blocked by the maternal anti-HBs as reported in animal experiments [24]. Such a phenomenon also occurs in vaccination of infants with hepatitis A vaccine in the presence of maternal antibody, in which antibody response substantially increases following an additional vaccine dose given six months after completion of the primary vaccination [25]. Therefore, the inhibition of high titer maternal anti-HBs on the immune response to the vaccine reported previously [12] may also be transient and the current vaccination schedule against hepatitis B may induce potent immune response in a relatively slow manner in the presence of high titer maternal anti-HBs. Additionally, higher-dose hepatitis B vaccine may overcome the inhibition of maternal anti-HBs on the immune response since the immunogenicity of three doses of 10 µg-vaccine on 0-, 1-, and 6-month schedule was comparable in Brazilian neonates with or without maternal anti-HBs [26].

Given the efficient transplacental transfer and higher levels of anti-HBs in infants than in mothers, it is reasonable to assume that infants of mothers with positive anti-HBs are immune to HBV infection in the early life. The results that passively acquired maternal anti-HBs in the infants efficiently bound patient-derived HBsAg (Figure 4) support this assumption. On the other hand, since the maternal anti-HBs in infants does not impair the long-term immunogenicity of hepatitis B vaccine as demonstrated in the present study, we consider that, unlike many other vaccines that are not administrated until several months or even longer after birth because of the interference of maternal antibodies on the active immune response, the current hepatitis B vaccination schedule for infants will be still effective even in the future when most newborns are anti-HBs positives due to the transplacental transfer of maternal anti-HBs.

## References

[1] But DY, Lai CL, Lim WL, Fung J, Wong DK (2008). Twenty-two years follow-up of a prospective randomized trial of hepatitis B vaccines without booster dose in children: final report.. Vaccine.

[2] Poovorawan Y, Chongsrisawat V, Theamboonlers A, Leroux-Roels G, Kuriyakose S (2011). Evidence of protection against clinical and chronic hepatitis B infection 20 years after infant vaccination in a high endemicity region.. J Viral Hepat.

[3] World Health Organization (2009). Hepatitis B vaccines.. Wkly Epidemiol Rec.

[4] Zhang S, Li RT, Wang Y, Liu Q, Zhou YH (2010). Seroprevalence of hepatitis B surface antigen among pregnant women in Jiangsu, China, 17 years after introduction of hepatitis B vaccine.. Int J Gynaecol Obstet.

[5] Siegrist CA (2001). Neonatal and early life vaccinology.. Vaccine.

[6] Siegrist CA (2003). Mechanisms by which maternal antibodies influence infant vaccine responses: review of hypotheses and definition of main determinants.. Vaccine.

[7] Glezen WP (2003). Effect of maternal antibodies on the infant immune response.. Vaccine.

[8] Bell BP, Negus S, Fiore AE, Plotnik J, Dhotre KB (2007). Immunogenicity of an inactivated hepatitis A vaccine in infants and young children.. Pediatr Infect Dis J.

[9] Johansson E, Istrate C, Charpilienne A, Cohen J, Hinkula J (2008). Amount of maternal rotavirus-specific antibodies influence the outcome of rotavirus vaccination of newborn mice with virus-like particles.. Vaccine.

[10] Saffar MJ, Ajami A, Khalilian AR, Saffar H (2009). The impact of maternal measles-rubella immunization on the 12-month-old infant's immune response to measles-mumps-rubella vaccine immunogenicity.. Eur J Clin Microbiol Infect Dis.

[11] Kurubi J, Vince J, Ripa P, Tefuarani N, Riddell M (2009). Immune response to measles vaccine in 6 month old infants in Papua New Guinea.. Trop Med Int Health.

[12] Hu Y, Wu Q, Xu B, Zhou Z, Wang Z (2008). Influence of maternal antibody against hepatitis B surface antigen on active immune response to hepatitis B vaccine in infants.. Vaccine.

[13] Zhou J, Hu Y, Liu Q, Chen Q, Xu B (2007). Analysis of birth defects based on population survey in Jiangsu Province.. Jiangsu Medical Journal.

[14] Shokrgozar MA, Shokri F (2002). Subtype specificity of anti-HBs antibodies produced by human B-cell lines isolated from normal individuals vaccinated with recombinant hepatitis B vaccine.. Vaccine.

[15] Wang L, Lin SJ, Tsai JH, Tsai CH, Tsai CC (2005). Anti-hepatitis B surface antigen IgG1 subclass is predominant in individuals who have recovered from hepatitis B virus infection, chronic carriers, and vaccinees.. Med Microbiol Immunol.

[16] Heijtink RA, Kruining J, Weber YA, de Man RA, Schalm SW (1995). Anti-hepatitis B virus activity of a mixture of two monoclonal antibodies in an “inhibition in solution” assay.. Hepatology.

[17] van Nunen AB, de Man RA, Heijtink RA, Vossen AC, Schalm SW (2002). Passive immunization of chronic hepatitis B patients on lamivudine therapy: a feasible issue?. J Viral Hepat.

[18] Simister NE (2003). Placental transport of immunoglobulin G.. Vaccine.

[19] Roopenian DC, Akilesh S (2007). FcRn: the neonatal Fc receptor comes of age.. Nat Rev Immunol.

[20] Ayoola EA, Johnson AO (1987). Hepatitis B vaccine in pregnancy: immunogenicity, safety and transfer of antibodies to infants.. Int J Gynaecol Obstet.

[21] Hartter HK, Oyedele OI, Dietz K, Kreis S, Hoffman JP (2000). Placental transfer and decay of maternally acquired antimeasles antibodies in Nigerian children.. Pediatr Infect Dis J.

[22] Esteban JI, Genesca J, Esteban R, Hernandez JM, Seijo G (1986). Immunoprophylaxis of perinatal transmission of the hepatitis B virus: efficacy of hepatitis B immune globulin and hepatitis B vaccine in a low-prevalence area.. J Med Virol.

[23] Xu ZY, Duan SC, Margolis HS, Purcell RH, Ou-Yang PY (1995). Long-term efficacy of active postexposure immunization of infants for prevention of hepatitis B virus infection. United States-People's Republic of China Study Group on Hepatitis B.. J Infect Dis.

[24] Weeratna RD, Brazolot Millan CL, McCluskie MJ, Siegrist CA, Davis HL (2001). Priming of immune responses to hepatitis B surface antigen in young mice immunized in the presence of maternally derived antibodies.. FEMS Immunol Med Microbiol.

[25] Dagan R, Amir J, Mijalovsky A, Kalmanovitch I, Bar-Yochai A (2000). Immunization against hepatitis A in the first year of life: priming despite the presence of maternal antibody.. Pediatr Infect Dis J.

[26] Junqueira AL, Tavares VR, Martins RM, Frauzino KV, da Costa e Silva AM (2011). Presence of maternal anti-HBs antibodies does not influence hepatitis B vaccine response in Brazilian neonates.. Mem Inst Oswaldo Cruz.

